# Mechanical transduction of cytoplasmic-to-transmembrane-domain movements in a hyperpolarization-activated cyclic nucleotide–gated cation channel

**DOI:** 10.1074/jbc.RA118.002139

**Published:** 2018-06-23

**Authors:** Christine Gross, Andrea Saponaro, Bina Santoro, Anna Moroni, Gerhard Thiel, Kay Hamacher

**Affiliations:** From the ‡ Computational Biology and Simulation Group,; ‖ Membrane Biophysics, Department of Biology, Technische Universität Darmstadt, 64287 Darmstadt, Germany,; the § Department of Biosciences, University of Milan, 20133 Milan, Italy, and; the ¶ Department of Neuroscience, Columbia University, New York, New York 10032

**Keywords:** potassium channel, protein conformation, computational biology, cyclic AMP (cAMP), protein dynamic, anisotropic network model, cAMP dependent gating, HCN1 channel, linear response theory

## Abstract

Hyperpolarization-activated cyclic nucleotide–gated cation (HCN) channels play a critical role in the control of pacemaking in the heart and repetitive firing in neurons. In HCN channels, the intracellular cyclic nucleotide–binding domain (CNBD) is connected to the transmembrane portion of the channel (TMPC) through a helical domain, the C-linker. Although this domain is critical for mechanical signal transduction, the conformational dynamics in the C-linker that transmit the nucleotide-binding signal to the HCN channel pore are unknown. Here, we use linear response theory to analyze conformational changes in the C-linker of the human HCN1 protein, which couple cAMP binding in the CNBD with gating in the TMPC. By applying a force to the tip of the so-called “elbow” of the C-linker, the coarse-grained calculations recapitulate the same conformational changes triggered by cAMP binding in experimental studies. Furthermore, in our simulations, a displacement of the C-linker parallel to the membrane plane (*i.e.* horizontally) induced a rotational movement resulting in a distinct tilting of the transmembrane helices. This movement, in turn, increased the distance between the voltage-sensing S4 domain and the surrounding transmembrane domains and led to a widening of the intracellular channel gate. In conclusion, our computational approach, combined with experimental data, thus provides a more detailed understanding of how cAMP binding is mechanically coupled over long distances to promote voltage-dependent opening of HCN channels.

## Introduction

Of an estimated 200 genes encoding ion channels in mammals, hyperpolarization-activated cyclic nucleotide–gated cation (HCN)^2^ channels are the only channels that open on membrane hyperpolarization but conduct a depolarizing inward current (1, 2). HCN channels are also the only voltage-gated channels regulated by the direct binding of cyclic nucleotides. By virtue of these properties, HCN channels play unique and essential roles in a variety of physiological processes, the most important being the generation of spontaneous electrical activity in the heart and the regulation of synaptic transmission in the brain (1–3). These channels, which are present in humans in the four isoforms HCN1–4, share the general architecture of voltage-gated K^+^ channels (3). Their monomers are built of six transmembrane domains (TMDs) of which the 4th TMD (S4) comprises the voltage sensor, and the last two TMDs (S5–S6) contribute to the ion-conducting pore (4). A specific feature of HCN channels is the presence of a cyclic nucleotide–binding domain (CNBD) at their cytosolic C termini. HCN channels are activated by membrane hyperpolarization, and this voltage-dependent activation is further modulated in an allosteric manner by binding of cyclic nucleotides to the CNBD (5). As a result of cAMP binding, the voltage dependence of channel opening is shifted to lower (less hyperpolarized) potentials. Thus, an intracellular increase in the cytosolic concentration of cAMP causes an earlier membrane depolarization and hence an acceleration of pacemaking (5).

A full understanding of the allosteric nature of HCN channel regulation by voltage and ligands requires insights into the mechanism responsible for processing both regulatory signals in the context of the whole protein. One component in this scenario, the CNBD, has been well studied. Its structure was solved at atomic resolution for most HCN isoforms in the presence of cAMP and for HCN2 also in the absence of cAMP (6–8). These data show that this domain is built from an eight-stranded β-roll, which is connected to one α-helix on the N-terminal side (A-helix) and two additional α-helices on the C-terminal side (B- and C-helices). The cAMP-binding site is composed by two elements within the CNBD: the distal C-helix and the phosphate binding cassette; the latter is embedded within the β-roll and contains the short P-helix. The CNBD is connected to the channel pore via a helical domain, the C-linker, which in turn is composed of six α-helices (A′–F′). The C-linker is critical for the transmission of conformational information between the CNBD and the transmembrane portion of the channel (TMPC) and is thus responsible for the communication between the two domains in channel regulation.

Recently, Lee and MacKinnon (9) obtained the first high-resolution structure of the full-length HCN1 channel in the cAMP-free and cAMP-bound state using cryo-electron microscopy (cryo-EM). The voltage-sensitive domain (VSD) of HCN1 is positioned next to the pore domain of the same subunit (nonswapped), an arrangement similar to that of the closely related Eag1 (Kv10.1) and CNG channels (10, 11); this is different from the voltage-gated K^+^ channels (Kv channels) where the VSD (S1–S4) is positioned near the pore domain of the neighboring subunit (domain-swapped). This latter arrangement of the VSD correlates with a much shorter S4–S5 linker, which in HCN1 is significantly shorter than the stretch of ∼15 amino acids (AA) typical of Kv channels. Despite providing significant advances in our understanding of HCN channel structure–function relations, the seminal study by Lee and MacKinnon (9) left the question of how conformational information is transmitted between the CNBD and the transmembrane portion of the channel largely unanswered. Purification of the HCN1 protein in 0 mV conditions resulted in a channel locked in a closed conformation, independent of the cAMP-free or cAMP-bound configuration of the CNBD, preventing the required analysis of protein movements within the TMPC.

To address open questions on the conformational dynamics in HCN channels, here and elsewhere, we use linear response theory (LRT). This mechanical model, which was introduced by Ikeguchi *et al.* (12), can help to calculate the direction of conformational changes in a protein upon external perturbation, *e.g.* by ligand binding. LRT is a coarse-grained modeling technique (13) that requires much shorter computational times than molecular dynamics (MD) simulations to obtain insight into protein dynamics around the native state. Despite the simplicity, this approach still generates results that match very well with experimental data (14–17) or results from all-atom MD simulations. This also holds true for the analysis of membrane proteins in models where the membrane is neglected, in order to further simplify the mechanical model (18).

To understand the mechanical connections between the HCN channel CNBD and the TMPC (*i.e.* the six transmembrane domains and the connecting linkers), in a previous study we employed LRT to model a synthetic channel in which the HCN channel C-linker/CNBD was connected to the available structure of the TMPC of the Kv2.1 channel (19). This is because, at the time of the study, there was no structural information available yet on the HCN channel TMPC. The simulation predicted that release of cAMP from its binding site triggers a quaternary twist in the cytosolic portions of the four subunits in the channel tetramer (19). This prediction turned out to be in good agreement with the cryo-EM structures of HCN1, which similarly suggest the predicted quaternary twist in the cytosolic parts in response to cAMP binding/release (9).

With the new structural information available on the entire HCN1 channel, we now revisit the open questions on the gating mechanism of HCN channels. In particular, we want to understand how the information from cAMP binding is transmitted to the TMPC and how conformational changes in the cytosolic domain are related to gating movements. For example, what is the movement of the S4 domain in response to cAMP binding? What is the movement of the S6 domain in response to cAMP binding? How may cAMP binding favor opening of the channel gate?

Because the mechanical connections and the directions of conformational changes are not known for most proteins, including HCN channels, we recently developed a reference model for LRT (LRT null model) for a monomeric protein (17). This method provides a way to uncover the mechanical response in proteins in an unbiased and efficient manner. Thus, instead of using information derived from a “typical” interaction, a functionally relevant residue in a protein is subjected to a set of random perturbations from any possible direction. The resulting responses, in the form of residue displacements, can then be clustered according to the general directions of displacement they impose on the protein structure. In the following step, perturbations from different clusters can be evaluated with respect to the plausibility of the direction of perturbation, which can be judged by comparing the computational data with experimental results obtained with the same protein.

**Figure 1.**
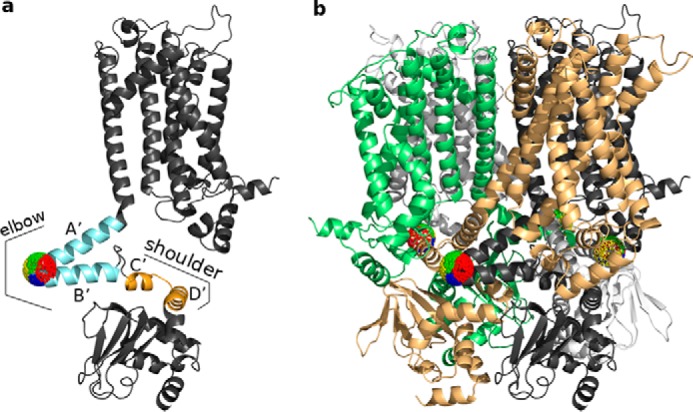
**LRT null model of cAMP-free HCN1 channel with clustering of different perturbation directions.** *a,* clustering of the different perturbation directions imposed on Ala-425 at the tip of the elbow (*light blue*) based on the displacement of the shoulder (*orange*). Both elements are part of the C-linker, which connects the CNBD to the S6 domain of the channel. For clarity, a single subunit of HCN1 is shown. Perturbation directions on a sphere around Ala-425 that belong to the same cluster are represented in the same color (*red, blue, yellow,* or *green*). *b,* clustering of the perturbation directions shown for all four subunits of HCN1. To illustrate contacts between individual subunits, the four monomers are shown in different colors. The *dark gray* color of the subunit in *b* corresponds to the same color in *a*.

In this study, the LRT null model was adjusted to work for the homotetrameric HCN1 channel. To identify the most plausible perturbation direction, we relied on previous experimental studies in which conformational changes in the cytosolic domains of HCN channels were monitored after binding of cAMP to the CNBD (8, 20, 35). Based on these studies, we decided to apply an external force at a single position located in the bend between the A′- and B′-helices of the C-linker. As stated above, the C-linker is in a strategic position (Fig. 1) as it connects the CNBD to the S6-helix of the pore, and thus, it is thought to transmit the conformational changes in the CNBD after cAMP binding/release to the pore (6, 20, 22).

We first show that all our modeling results are in good agreement with experimental data and thus confirm the assumption that the tip of the C-linker “elbow” (6) is an important position for the mechanical transduction of information from the CNBD to the HCN channel pore and vice versa. Next, we employ LRT analysis to predict the conformational changes in the orientation of the transmembrane helices, as well as the movement of the inner gate of HCN1, in response to cAMP binding. More specifically, we show how tilting movements in the transmembrane helices, predicted by the LRT model, generate two important outcomes implied by prior experimental studies: 1) an increase in the distance between the voltage sensor (S4) and the surrounding transmembrane domains; and 2) a widening of the intracellular channel gate. Altogether, the combination of a high-resolution structure and LRT analysis provides a valuable tool for uncovering the short- and long-range mechanical connections in HCN channels, which are relevant for their gating by ligands.

## Results

### C-linker movement after perturbation at the elbow

The C-linker is an important structure in HCN channels for the coupling of conformational changes in the CNBD, which are generated by cAMP binding, with the channel pore (23, 24). In all HCN structures available, the C-linkers are found tightly packed in the tetramer in the so-called “elbow on the shoulder” conformation in which the A′- and B′-helices (elbow) of one subunit interact with the C′- and D′-helices (shoulder) of the adjacent subunit (6, 9). Based on experimental data, which related channel activation of cyclic nucleotide–gated channels with structural features in the CNBD (20, 25, 26), a model was proposed in which, upon cAMP binding, the elbow moves with respect to the shoulder of the adjacent subunit in an overall centrifugal motion, away from the central axis of the channel, which causes a widening of the inner pore (27).

**Figure 2.**
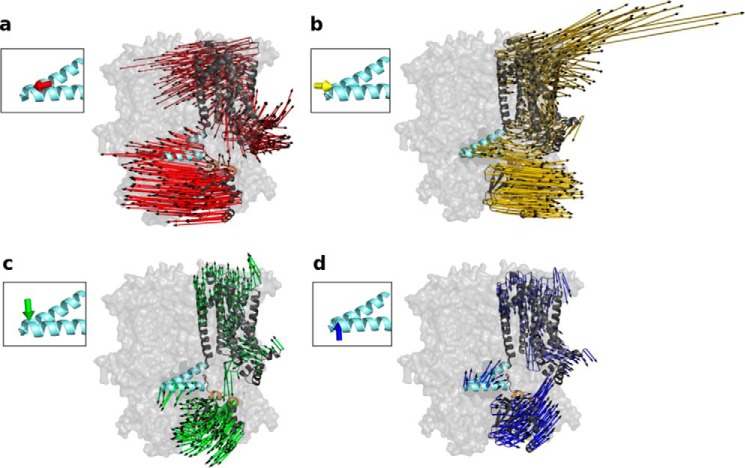
**Perturbation of the ANM from HCN1 in the cAMP-free form.** Perturbations at the tip of the elbow from four different clusters lead to distinct horizontal and vertical displacements of the C-linker. Displacements of HCN1 after perturbation at Ala-425 from one representative direction (*a–d*) for each cluster correspond to the coloring in Fig. 1. For clarity, the displacements are only shown for one subunit, which is highlighted in cartoon representation. The direction of force application is illustrated in the *insets*. The other subunits are illustrated in transparent surface representation. The *thin arrows* demonstrate the displacement of each residue. As in Fig. 1, the elbow is highlighted in *light blue*, and the shoulder is highlighted in *orange*.

To simulate the proposed C-linker movement, we perturbed the anisotropic network model (ANM) of cAMP-free HCN1 structure at the tip of the elbow (Ala-425 in the bend between the A′- and B′-helices) with forces from different directions. The elbow was chosen to perturb the protein because it seems to undergo a large conformational change during cAMP binding (27). To investigate which of these perturbation directions causes conformational changes that best match the experimentally observed C-linker movement, the force directions were first clustered into groups of similar displacement effects. Clustering, which is described in detail under “Experimental procedures,” was based on the effect of the elbow movement on the underlying shoulder, represented by residues 446–465 on the C′- and D′-helices. The shoulder domain was chosen for clustering because it is directly coupled to the elbow and hence responds in a direct manner to perturbations at the elbow (27). Clustering based on movements of all residues would be less meaningful as unstructured loop regions can move very randomly. The clustering of perturbation directions at the tip of the elbow, which induces different movements of the shoulder, is illustrated in Fig. 1. The displacements observed in response to one representative force direction for each of the four different clusters are shown in Fig. 2, where the coloring of the displacement vectors corresponds to the coloring of clusters in Fig. 1 (and in all following figures), and the length of the arrows is proportional to the size of the LRT displacements (rendered in arbitrary units).

**Figure 3.**
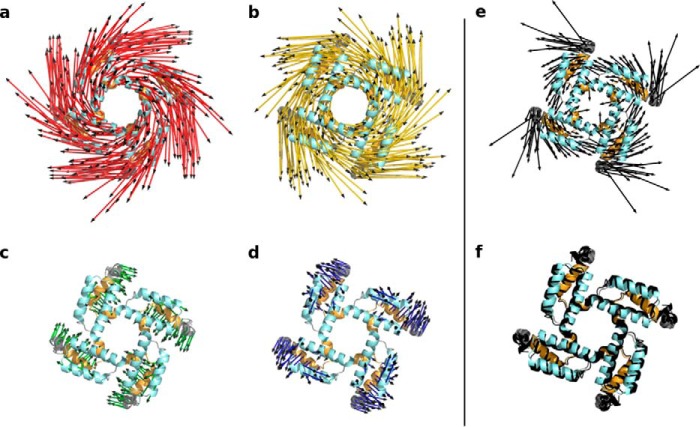
**Comparison of predicted and measured displacements of the C-linker in cAMP-free and cAMP-bound HCN1 channel.** Displacements of the C-linker of HCN1 after perturbation at Ala-425 are from one representative direction (*a–d*) for each cluster. Coloring corresponds to clusters in Figs. 1 and 2. *e,* experimentally observed displacement of the C-linker after superposition of the full-length cAMP-free and cAMP-bound HCN1 structures. Because the displacements are very small in the experimental structures, the *arrows* shown represent an arbitrary multiple of the actual displacement so as to allow to see the directions properly and compare them with the LRT displacements. *f,* superposition of the cAMP-free (*colored*) and cAMP-bound (*black*) HCN1 structures showing the actual displacements. In all visualizations the proteins are shown in top view from an extracellular perspective, so that the movements of all four subunits can be seen. The elbow again is highlighted in *blue,* and the shoulder is highlighted in *orange*. The remaining residues are colored in *gray*.

The data show in a side view perspective that horizontal (in the plane of the membrane) and vertical (perpendicular to the plane of the membrane) perturbations cause distinct displacements in the C-linker/CNBD domain as well as in the TMPC. Notably, the strongest displacements (*longer arrows*) throughout the protein are elicited by perturbations applied in a horizontal direction parallel to the plane of the membrane (Fig. 2, *a* and *b*). These perturbations (Fig. 2, *a* and *b, red* and *yellow arrows*) show the best match with the previously proposed model for C-linker movements in response to cAMP binding (20, 25, 26). The predicted iris-like movement of the C-linker of HCN1 can indeed be seen in the simulations. A top view of the C-linker shows an iris-like motion as a result of the horizontal displacements of the elbow (Fig. 3, *a* and *b*). Vertical perturbations, as represented by the force direction vectors in the other two clusters, do not induce such movements (Fig. 3, *c* and *d*).

To further validate the conformational changes, which are predicted in our computational results, we used the high-resolution structures of HCN1 in the cAMP-free and cAMP-bound conformation for comparison (super-position, Fig. 3*f*) (9). Fig. 3*e* shows the position of the elbow and shoulder in the cAMP-free form. The *arrows* in Fig. 3*e* represent the movements in the protein, which are required for the transition from the experimentally determined cAMP-free into the cAMP-bound structure (9). A comparison between the simulated and experimental data shows that the displacement in Fig. 3*b* (*yellow arrows*) in which a force on the tip of the elbow is applied pushing toward the central axis of the protein (inward) reveals a very good match with the direction of the conformational changes of the C-linker observed after cAMP binding. As the tip of the elbow is pushed toward the central axis of the protein, the helices of the elbow and the underlying shoulder respond by all moving in the overall same direction away from the center in a centrifugal motion, but at different angles. This indeed generates an iris-like rotational movement, seen in the top view perspective of Fig. 3, *b* and *e,* as a counter-clockwise rotation.

Whereas the horizontal movement of the elbow in Figs. 2*b* and 3*b* recapitulates the experimentally determined iris-like transition of the C-linker in response to cAMP binding, it is important to note that in all HCN structures resolved thus far the C-linker is found in the resting (nonactive) position. Thus, in the full-length HCN1 structure (obtained at 0 mV), the depolarized position of VSD always locks the channel in a closed state, whether cAMP is present or not. This is likely to impose a severe limitation on the range of movement the C-linker is able to undergo (see Fig. 3, legend). Similarly, it has been suggested that in all available crystal structures for the cAMP-bound C-linker/CNBD fragment, the C-linker is found in a resting state, as inferred by the presence of a set of saline bridges in the structure, which are postulated to break in the open state of the HCN channel (20, 26). Our modeling data are able to simulate a much wider range of C-linker movements independent of the presence or absence of these critical salt bridges and to document these effects of movements on the rest of the protein. Although arbitrary, such a wider range of movements is likely to provide a useful representation of the scope of motions found in the actual protein.

Thus, the simulations also show how a horizontal force applied in the outward direction (*red arrows*) causes a displacement in the opposite direction (Figs. 2*a* and 3*a*). This displacement likely reflects the direction of conformational changes the protein may undergo upon cAMP release, and indeed, very similar residue displacements are observed when the same force is applied on the ANM of the cAMP-bound HCN1 structure (Fig. S1). Based on these results, we proceeded to further analyze all conformational changes under the assumption that displacements elicited by the inwardly directed horizontal force applied to the elbow of the cAMP-free HCN1 structure (as represented by the *yellow arrows* in Figs. 2 and 3) reflect the structural changes that are induced by cAMP binding in the actual HCN channel protein.

### Effects of C-linker movement on the CNBD

**Figure 4.**
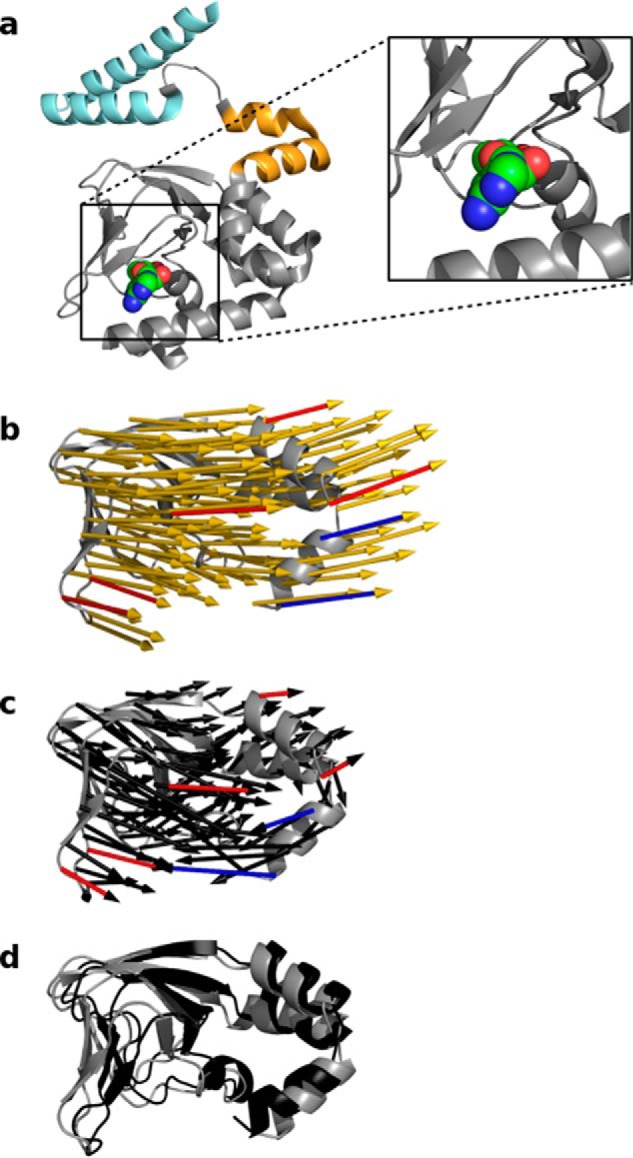
**Comparison between predicted and measured displacements of CNBD in cAMP-free and cAMP-bound HCN1 channel.** *a,* cAMP-bound HCN1 structure (residue 401–608, cytosolic domain) showing the orientation of cAMP within the binding pocket. The HCN1 structure is shown in cartoon representation with elbow and shoulder of the C-linker colored according to Fig. 1. The cAMP molecule is highlighted by *sphere* representation. *b–d,* comparison of the displacements in the CNBD (residues 480–586) after perturbing the elbow from the most realistic direction (*b, yellow arrows*) to the experimentally resolved displacement between the cAMP-free (*gray*) and cAMP-bound (*black*) HCN1 structure (*c, black arrows*; *d, superposition*). The length of the *arrows* was chosen such that the length in *b* and *c* are similar; the absolute length has no quantitative meaning. The *red* and *blue arrows* highlight exemplary displacements, which are similar or different between the computational model and the experimental data, respectively. The experimental data ±cAMP are from Ref. 9. For clarity, only one subunit of HCN1 is shown.

The modeling data suggest that a horizontal movement of the C-linker transmits the conformational changes in the CNBD, which originate from cAMP binding, toward the TMPC. Because LRT and elastic network modeling approximate the mechanics of a protein around a stable configuration, we must assume that any movement, which is induced by a local perturbation, is bidirectional. This means that any force, which leads to a distinct structural response, must be reproduced by the same structural perturbation. Using this logic, we can assume that the same perturbation at the elbow, which mimics cAMP binding, should also cause a realistic conformational change in the opposite direction, namely toward the cAMP-binding domain. To test this prediction, we analyzed the effect of the C-linker movement after the perturbation mimicking cAMP binding (Fig. 2*b*, *yellow arrows*) on the CNBD. The computational prediction can be compared with the conformational changes observed in the experimental HCN structures in the presence and absence of cAMP (6, 8). Fig. 4*a* illustrates cAMP within the binding pocket of the CNBD. The *yellow arrows* in Fig. 4*b* illustrate the predicted movements in the CNBD in response to a force on the elbow, which mimics cAMP binding. The corresponding movements from experimental data are represented by *black arrows* in Fig. 4*c*. The relative movement of the domains can be appreciated from a superposition of cAMP-free and cAMP-bound structures (Fig. 4*d*). Comparison of both data sets (Fig. 4, *b* and *c*) shows a similarity but also differences. The similarity is that cAMP binding induces *in silico* as well as in the experimental structures an overall outward movement of the CNBD, driven by the outward movement of the shoulder described above; representative arrows which point in the same direction in both structures are highlighted in *red*. Also in this case, the computational model recapitulates the experimental data. Differences between the two predictions occur mostly within the C-helix, where the directions of the theoretical and experimentally predicted displacements point in opposite directions (highlighted by the *blue arrows*; Fig. 4, *b* and *c*). The deviations between the two approaches for the C-helix come as no surprise; the experimental data were obtained in the presence and absence of cAMP, respectively, so that the conformational changes reflect the sum of ligand binding/release plus the subsequently triggered conformational changes in the CNBD, which include folding of the C-terminal portion of the C-helix and formation of the P-helix (8). The computational data, however, only capture the conformational changes in the protein occurring as a result of cAMP binding. Therefore, it should be expected that the computational and the experimental structures differ in some of the elements that make direct contact with cAMP, because the latter may undergo conformational changes during ligand binding (8).

### Effects of cAMP binding on conformations in the transmembrane portion of the channel

#### 

##### Coupling of C-linker to S4–S5 linker

**Figure 5.**
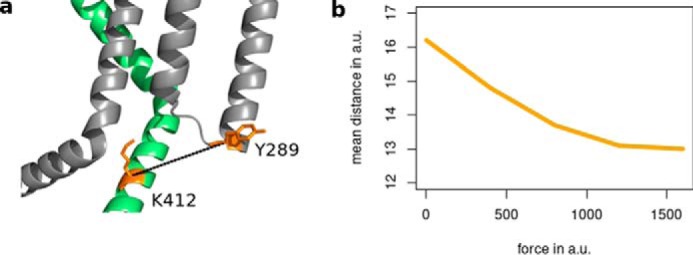
**Simulation predicts that the C-linker and the S4–S5 linker move closer together after cAMP binding.** *a,* visualization of the distance between Tyr-289 of one subunit (*gray*) and Lys-412 of the neighboring subunit (*lime green*). *b,* computed distance between the CA positions of Tyr-289 and Lys-412 of two neighboring subunits after perturbation at Ala-425 with forces of increasing strengths (*yellow cluster* in Figs. 2 and 3). Both the forces as well as the distances are given in arbitrary units (*a.u.*) and thus can only show a trend.

Several studies have highlighted a role for the HCN channel VSD in the allosteric regulation of cAMP affinity for the CNBD (28–31). These effects could be potentially explained by the suggested interaction between the S4–S5 linker and the C-linker (32). Several studies have proposed that the relative orientation of these two domains changes during channel gating (33, 34). In a recent study, the distance between an AA of the S4–S5 linker (Phe-359) and the A′-helix in the C-linker of a HCN channel from sea urchin sperm, called SpHCN, was monitored using transition metal ion FRET (21). The measurements showed that cAMP binding reduced the distance between the S4–S5 linker and the C-linker. To compare these experimental results with the predictions from our computational study, we assessed the distance between AAs Tyr-289 and Lys-412 in the HCN1 ANM structure, the former position corresponding to Phe-359 in spHCN and the latter to the center of the A′-helix in the C-linker. The data in Fig. 5 show that the LRT calculation exhibits the same conformational change predicted from the experimental study; indeed, application of an appropriately directed force to the tip of the elbow, *i.e.* a force that triggers the conformational changes of cAMP binding (*yellow arrows* in Figs. 2 and 3), results in a reduced distance between the C-linker and the S4–S5 linker. This distance decreases in an exponential fashion with the strength of the force. It is important to note that the LRT only provides qualitative results in terms of the directions of displacements in a protein. For this reason, we can only show whether the distance decreases or increases; the actual magnitudes of displacements cannot be computed, and thus, the distances are only given in arbitrary units.

The results of these experiments suggest that a cAMP-induced conformational change in the CNBD is mechanically transmitted via the C-linker to the S4–S5 linker. A potential physical interaction between the two domains provides a plausible mechanism for a reciprocal communication between the cytosolic domain and the TMPC.

##### Coupling of C-linker to VSD and pore module

**Figure 6.**
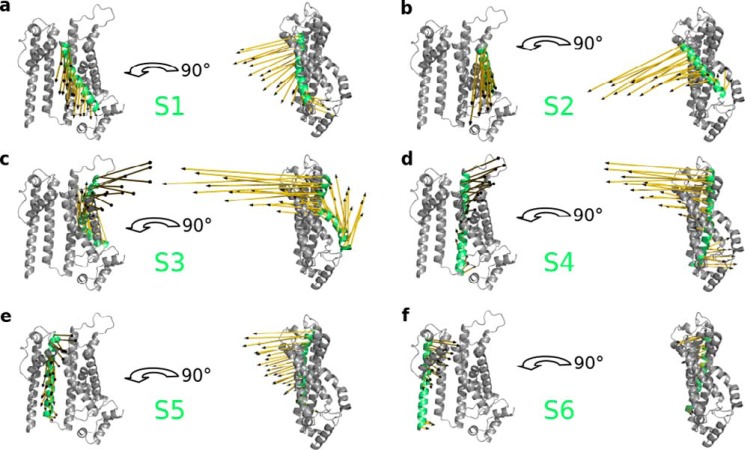
**Simulation predicts tilting movements of transmembrane helices S1–S6 after cAMP binding.** Displacements of S1 to S6 helices of the TMPC (*a–f*) after perturbing the elbow of the C-linker from the most realistic perturbation direction. The corresponding helix is highlighted and labeled in *lime green,* and the displacement is visualized as *yellow arrows*. For clarity, only one subunit (residue 94–402) is shown from the front and side views.

The experimental structures of full-length HCN1 reveal hardly any differences in the TMPC (VSD and pore module) between the cAMP-free and cAMP-bound form. This would seem to imply that the movement of the C-linker only generates, at least in the absence of a membrane voltage, subtle changes in the TMPC. To obtain more information on how cAMP can then so effectively modulate the voltage-dependent gating of the channel, we visualized the displacements in HCN1 after perturbation at Ala-425 from the direction that simulates cAMP binding (*yellow arrows* in Figs. 2 and 3). The resulting displacements of the six helices S1–S6 of the TMPC are illustrated in Fig. 6 in a front and side view and in Fig. S2 from a top and bottom perspective. This analysis shows that the C-linker movement has indeed a distinct effect on the TMPC. All six helices undergo a tilting in the sense that the upper parts move into the opposite direction from the lower parts (Figs. 6 and Fig. S2, *a–f*). It is worth noting that the angles and directions of movement are somewhat different for each TMD. The results of this analysis imply that cAMP binding may facilitate voltage-dependent opening of the channel by inducing a differential tilting like movement of the TMDs. The modeling data underscore a remarkable degree of movement at the extracellular end of the TMDs and interconnecting loops. This observation may provide a potential explanation for prior experimental studies, which have demonstrated a critical role for these elements in the modulation of HCN channel gating (see “Discussion”).

**Figure 7.**
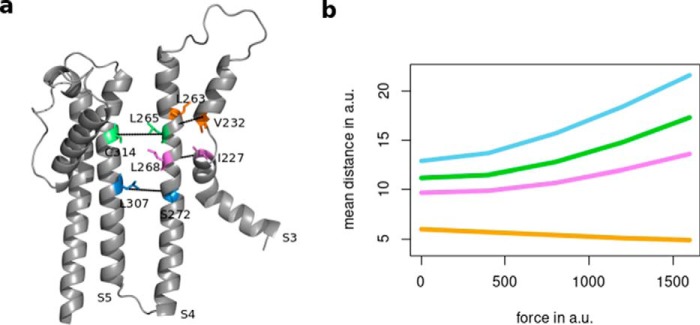
**cAMP binding predicts a local increase in the distances between S4 and surrounding transmembrane domains.** *a,* HCN1 monomer with reference amino acids in S3, S4, and S5. *b,* computed distances between these amino acids in the same subunit after perturbation of Ala-425 with forces of increasing strengths (*yellow cluster* in Figs. 2 and 3). The colors in *b* cross-reference with colors in *a*. Forces as well as the distances are again given in arbitrary units (*a.u.*).

Our computational data, however, show no indication for a vertical displacement of the transmembrane helices, including S4, in response to protein perturbations mimicking cAMP binding. This, of course, does not rule out that vertical displacements of S4, similar to what is seen in Kv channels, may occur in HCN channels in response to voltage. Indeed, a vertical translocation of S4 in response to voltage was suggested by an early study using cysteine accessibility methods in the sea urchin HCN channel (35). Later studies employing similar methods in the mammalian HCN1 channel, however, have cast some doubt on this simple model (36, 37). These studies demonstrate that the intracellular region of S4 that displays state-dependent modification is much larger than the state-dependent extracellular S4 region and that the observed modification rate shifts are much more pronounced for residues at the intracellular compared with the extracellular end of S4 (36, 37). These incongruences have been variously explained either by postulating an “unwinding” of the S4-helix upon hyperpolarization, such that the lower end is able to undergo a vertical translation while the upper end remains relatively stable (36). As an alternative scenario, it has been postulated that a rearrangement in the transmembrane segments surrounding S4 may cause formation or collapse of a water-filled internal “gating” canal in response to negative or positive voltages, respectively. As a consequence, the shape of the membrane field surrounding the S4 segment would correspondingly be altered (37). In light of the latter hypothesis, we tested the possibility that cAMP binding might alter gating by affecting the width of this internal aqueous gating canal. We therefore measured the distance of reference residues within S4 with partner residues on S5 and S3 (Fig. 7*a*). In both previous studies, the residues in the mouse HCN1 channel corresponding to Leu-265 and Ser-272 in human HCN1 (Fig. 7) became accessible to sulfhydryl-modifying methanethiosulfonate reagents upon hyperpolarization (36, 37). Our present data show that application of a force on the elbow, in a direction which simulates cAMP binding, affects the distance between S4 and the surrounding transmembrane domains (Fig. 7*b*). Again, distances can only be given in arbitrary units, and thus, we only focus on whether the distances between residues of interest decrease or increase. Increasing force augments a separation between S4 and S5 and between S4 and the lower segment of S3 (S3a). Together with the experimental data, this suggests that negative voltage and cAMP binding may act in the same manner in that they open up an internal gating canal. This could provide an explanation for the allosteric function of ligand binding and voltage toward the opening of HCN channels.

##### Coupling of C-linker to the inner gate

**Figure 8.**
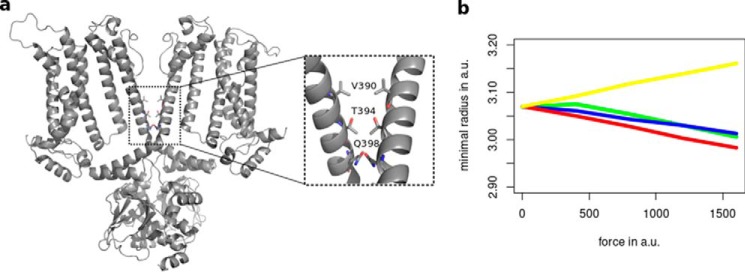
**Only a horizontal displacement of the elbow causes widening of inner channel gate.** *a,* location of inner gate (*inset*) in the global structure of HCN1. *b,* development of minimal inner gate radii in the region from Val-390 to Gln-398 with forces of increasing strengths acting on Ala-425. Force direction is represented according to the color coding used in Figs. 2 and 3. Both the forces applied and the minimal radii are given in arbitrary units (*a.u.*) as the LRT model only provides a trend for protein movements, in a qualitative manner but not quantitatively. Upon perturbations from directions of the *yellow cluster*, a widening of the inner gate can be observed, whereas for perturbations from other directions, the inner gate becomes narrower.

The current view on HCN channel activation is that they undergo a voltage-dependent transition from a resting state to a nonconductive active state before opening in a final voltage-independent transition. The latter step is allosterically modulated by cAMP binding to the CNBD, which in turn favors the opening transition in the pore via C-linker movements (38, 39). It has been proposed that cAMP binding stabilizes the open state by a rotational movement of the C-linkers and a consequent widening of the inner channel gate (20, 26). Having determined that a lateral pushing force on the C-linker elbow results in transmission of conformational forces to the TMPC in our model, we further proceeded to examine the associated changes in the radius of the inner gate. The respective region, comprising residues 390–398 of the S6-helix at the constriction of the inner gate, is shown in Fig. 8*a*. Fig. 8*b* shows the development of the minimal inner gate radius with forces of increasing strengths acting on Ala-425. The analysis shows that only perturbations from directions of the yellow cluster, which simulates cAMP binding, lead to a relative pore widening. Perturbations from all other directions cause in contrast a narrowing of the inner gate.

The results of this analysis further supports the assumption that the movements in the protein, which are caused by a force direction from the yellow cluster, mimic the conformational changes that take place after cAMP binding. Thus, the cAMP-dependent rearrangements, which originate in the CNBD, are ultimately transmitted to the TMPC in such a manner as to favor the opening of the inner gate. In contrast, gate opening is opposed and fully reversed by the conformational changes, which are induced by cAMP release from the CNBD (Figs. 2*a* and 3*a*, *red arrows*).

## Discussion

The current challenge in understanding HCN channel function is to integrate information from static, high-resolution structures with a wealth of functional data on the dynamics of channel gating. Here, we show that LRT provides an alternative route to understand how ligand binding in the CNBD is related to channel gating in the transmembrane part of the protein. This coarse-grained computational approach offers less molecular details than conventional MD simulations and also ignores the impact of the membrane on the protein of interest. But despite these simplifications the LRT approach still provides valuable insights into the mechanic connections within the protein. Based on the available high-resolution HCN1 structure (9), the LRT computations reproduce without any bias (17) many of the conformational changes, which were observed in structural experiments in response to cAMP binding. Our approach is based on the assumption that movements in the elbow of the C-linker are mechanically connecting, in a bidirectional manner, conformational changes in the CNBD and in the TMPC. This model, which was originally proposed on the basis of experimental data (20, 29, 30), is perfectly reproduced by the computational data. The simulations show that only a horizontal inwardly directed force applied to the tip of the elbow causes the same rotation and iris-like opening of the C-linker domain, which had been first proposed based on functional experiments (20, 26) and then observed in structural experiments on HCN channels in response to cAMP binding (9). In the LRT computations, this rotational movement propagates in the direction of the TMPC, where it causes a tilting of the TMDs and a small but distinct widening of the inner gate at the cytoplasmic end of the pore. This movement is overall consistent with the conformational changes between the cAMP-free and cAMP-bound HCN1 structure, which also suggest a concerted rotation of the C-linker and displacement of the S6-helix in favor of channel opening (9).

Our computational results furthermore show that the same horizontal force application on the elbow also propagates in the opposite direction toward the CNBD. There it generates distinct conformational rearrangements in the CNBD. The conformational changes, which occur in response to an imposed inward directed force at the tip of the elbow, are overall very similar to those observed in response to cAMP binding to the CNBD in experimental studies (8).

Collectively, the results of these analyses underscore a very good agreement between predictions from the computational simulation and experimental data. With this support for the predictive power of the computational method, we can now address the question of how cAMP binding may facilitate opening of HCN channels. The simulation data show that the rotation and iris-like opening of the cytosolic domain, which is triggered by cAMP binding, propagate into the TMPC, where it causes a distinct tilting of the TMDs. A close scrutiny of the S4 domain shows that a cAMP induced movement causes only a lateral but no vertical displacement of the voltage sensor. Such a finding is consistent with previous studies, which have suggested that a vertical movement of the S4 domain may not be of central importance in the gating of HCN channels (37) but rather that the voltage dependence of HCN channels may be modulated by a rearrangement of the TMDs surrounding the S4 domain. A central argument in this scenario is that the formation, or collapse, of a water-filled crevice (internal gating canal) could alter the shape of the electrical field around the S4 segment (36, 37). In light of this model, it was very intriguing to find that the computational data highlight a general tilting type movement of all TMDs in response to cAMP binding. The analysis of distances between critical residues in S4 with respect to reference residues in S3 and S5 shows that cAMP binding indeed augments a space between the voltage sensor domain and its surrounding helices. This effect of cAMP binding might lower the energy barrier for the opening of an internal gating canal and in this way facilitate HCN activation by negative voltages (37). Whereas our computational data do not rule out the possibility that S4 may undergo a vertical translational movement in response to voltage, as some other authors have suggested (35), the results certainly lend support to the notion that important lateral displacements may occur in S4 and its surrounding TMDs during HCN channel gating. Our LRT calculations also show that, in response to cAMP binding, the A′-helix of the C-linker moves closer to the S4–S5 linker. Several previous experimental studies have speculated that such a movement could similarly be important for the facilitated activation of HCN channels in the presence of cAMP (32–34).

A second central result of the simulations is that the rearrangement of the TMDs, which is triggered by movements in the CNBD and C-linker, causes a progressive widening of the inner gate at the intracellular end of the channel pore. Very intriguingly, we find that such widening of the inner gate is only achieved upon a horizontal inward movement of the elbow, which follows cAMP binding; the entire process is reversed by a movement in the opposite direction, which is presumably triggered by cAMP release from its binding site. It is also important to note, in this context, that a movement of the elbow in the vertical direction has only a minor impact on the width of the inner gate and that there is little difference between an upward or downward movement. The results of these data therefore suggest that an upward or downward movement of the C-linker elbow is not immediately apparent in cAMP-mediated channel gating.

The conformational change at the cytoplasmic end of the HCN1 channel pore is a potential mechanism by which cAMP binding may modulate the voltage-dependent opening of HCN channels, as was also suggested on the basis of previous experimental data (9, 20). Because LRT only provides a qualitative and not quantitative indication about the trend of protein movement, we cannot gauge whether the induced widening observed in the simulation would be sufficient to open the intracellular channel gate. Other mechanisms may very well be at play, including additional movements induced by changes in voltage across the membrane, which are not captured in our simulation. Nonetheless, the bidirectional effect on C-linker dynamics in response to cAMP binding/release represents a perfect reversible mechanism for the long-distance modulation of HCN gating.

Finally, on a more speculative note, the LRT simulation revealed a remarkably wide scope of motion at the extracellular end of the HCN1 protein TMDs. Although very little is known about the role of movement at the top of the TMDs and interconnecting loops (S1–S2, S3–S4, and S5–S6, including the selectivity filter), several studies have implicated these elements in the modulation of HCN channel gating. Residues in the S1–S2 loop influence the gating kinetics of HCN channels, and thus contribute to determine the different properties of the HCN1, HCN2, and HCN4 channel isoforms (40, 41). Variations in the sequence and length of the S3–S4 loop prominently modulate the voltage dependence of HCN1 channels (42, 43). More recently, a mutation located at the extracellular end of S6, identified in a Brugada syndrome patient, was found to shift the voltage dependence of the HCN4 channel by nearly 10 mV (44). These results collectively suggest that critical interactions are likely to occur around the outer mouth of the HCN channel pore, which may regulate movement during HCN channel gating. Future simulations, coupled with experimental structure–function analysis, may be able to provide insights into this question, and the many other open questions still remaining on the dynamics in HCN channels.

## Experimental procedures

### Linear response theory

This mechanical model (12) predicts conformational changes in a protein upon ligand binding. It is based on either the normal mode analysis (NMA) or elastic network models (ENMs), both of which extract collective motions in proteins (40, 45). NMA, which is based on traditional MD force fields, can be applied to all-atom structures as well as to reduced, coarse-grained networks of projected beads and springs. The alternative approach, the so-called ENMs (13), directly starts from a given (PDB) structure of a biomolecule. In the latter, the protein residues are represented by beads, and their covalent and noncovalent interactions are modeled as springs with corresponding spring constants. Interacting residues are defined by a cutoff threshold for spatial distances. Fluctuations of amino acids in ENMs can either be treated as isotropic, like in Gaussian network models, or as anisotropic, as in anisotropic network models (ANMs) (13, 41, 46). Because LRT is based on ANMs, it is able to predict the direction of a structural change of a protein upon ligand binding; ligand binding is in this case mimicked by an external force vector (12).

The translocation Δ*R⃗_i_* of atom *i* can be predicted by Equation 1,

(Eq. 1) $$\Delta {\vec{R}}_{i}≃\beta \;\cdot \;\sum_{j}{\langle \Delta {\vec{R}}_{i}\;\cdot \;\Delta {\vec{R}}_{j}\rangle }_{0}\;\cdot \;{\vec{f}}_{j}$$

where 〈Δ*R⃗_i_*·Δ*R⃗_j_*〉 represents the covariance matrix of the fluctuations of the protein in the ligand-free state, and *f⃗_j_* denotes the external force vector that mimics ligand binding. *B* is 1/*k_B_T* with the Boltzmann constant *k_B_*. The covariance matrix can be computed as the Moore-Penrose pseudoinverse (47, 48) of the Hessian matrix of an ANM (13).

### Structure preparation

All computations used the recently published cAMP-free structure of the HCN1 channel (PDB code 5U6O) (9) to simulate the effects of cAMP binding. The missing residues in the loop regions 201–202 and 243–251 of this cryo-EM structure were generated using MODELLER (version 9.15) employing the loop model function with fast loop refinement and restraint protein (49, 50). Afterward, the remodeled structure was energy-minimized using GROMACS (version 2016.1/2016.2) (51) during 25,000 steps with steepest descent *in vacuo* to solve possible clashes in or with the remodeled domains. As there were some tiny conformational changes in the C-terminal region after energy minimization in vacuum (CA root mean square deviation of 0.168 Å), the remodeled structure of residues 94–405 was combined with the original structure of residues 406–586, where no residues were missing. This was done by identifying overlapping residues in the C-linker region (AA 405 and 406.) Subsequently, the coordinates of residues 94–405 of one structure was merged with residues 406–586 of the second structure into one PDB file. This combined structure of HCN1 was used for further computations.

The same procedure was carried out with the cAMP-bound form of HCN1 (PDB code 5U6P) to study the actual motions, which are occurring by cAMP binding. After remodeling of the missing loop regions, no conformational changes occurred in the C-terminal region. The resulting energy-minimized structure was used for further comparative computations.

### Perturbation of HCN1 structure by external forces

The curated cAMP-free HCN1 structure was reduced to a heterogeneously parameterized anisotropic network model (ANM) (13) with a cutoff for connected residues of 13 Å, which was shown in a previous study to be a good choice (17). For intra-chain interactions between amino acids, we used the matrix of spring constants from Miyazawa and Jernigan (52), and for inter-chain interactions, we used the matrix for spring constants from Keskin *et al.* (53) as derived in Ref. 54. The ANM, which is illustrated in Fig. S3, was afterward perturbed at Ala-425 at the tip of the elbow of the C-linker from 1000 different directions using 1000 external force vectors with a force strength in arbitrary units. Therefore, the LRT null model introduced in a previous study for a small monomeric protein (17) was adjusted to a homotetrameric protein like HCN1. To account for the rotational symmetry of the tetramer, Ala-425 of each subunit was simultaneously perturbed by the same 1000 (also rotated) force directions. Afterward, clustering of the random force directions into four clusters was done based on the induced displacements of residues 446–465 (C′ and D′ helices), which represent the “shoulder” of the C-linker. The displacement vectors of the selected residues after perturbation were clustered by applying the *k*-means algorithm from Hartigan and Wong (55). The corresponding force directions were accordingly clustered and visualized by different colors in Fig. 1. Robustness of clustering was proved by a 1000-fold repetition of the clustering approach (17).

With this strategy, we investigated possible influences of the movement of the elbow (A′- and B′- helices) of the C-linker toward the shoulder of the C-linker. The decision for four clusters was based on the comparison of log values of maximal within-cluster sum of squares (maximum within ss) from *k*-means clustering as a function of the number of clusters, which was explained in detail in Ref. 17 (Fig. S4).

For each cluster, one representative force direction was chosen to determine the resulting displacements. Therefore, perturbations for each selected force direction were computed for repulsive forces with varying strengths to determine a trend for increasing force strengths.

### Computation of inner gate radii

Computation of the inner gate radii was done by using the program HOLE (56). The computations were restricted to the region from Val-390 to Gln-398. The values of minimal radii within this region were extracted from the HOLE output.

## Author contributions

C. G. data curation; C. G. formal analysis; C. G. visualization; C. G. and K. H. methodology; C. G., A. S., B. S., A. M., G. T., and K. H. writing-original draft; A. S., B. S., A. M., G. T., and K. H. conceptualization; A. M., G. T., and K. H. funding acquisition; K. H. resources; K. H. software; K. H. supervision.

## Supplementary Material

Supporting Information

## Footnotes

2 The abbreviations used are:

HCN: hyperpolarization-activated cyclic nucleotide–gated cation channel
AA: amino acids
ANM: anisotropic network model
CNBD: cyclic nucleotide–binding domain
MD: molecular dynamics
TMD: transmembrane domains
TMPC: transmembrane portion of the channel
VSD: voltage-sensitive domain
ENM: elastic network model
NMA: normal mode analysis
PDB: Protein Data Bank
LRT: linear response theory.
