# Supplementary material for: Mechanical transduction of cytoplasmic-to-transmembrane-domain movements in a hyperpolarization-activated cyclic nucleotide–gated cation channel
Source: J Biol Chem. 2018 Jun 23;293(33):12908–18. doi: 10.1074/jbc.RA118.002139 (PMC6102142; doi:10.1074/jbc.RA118.002139)
Supplement: Supporting Information [file supp_293_33_12908__index.html]

Mechanical transduction of cytoplasmic-to-transmembrane-domain movements in a hyperpolarization-activated cyclic nucleotide–gated cation channel — mechanics in HCN channel — Mechanical transduction of cytoplasmic-to-transmembrane-domain movements in a hyperpolarization-activated cyclic nucleotide–gated cation channel — Mechanics in HCN channels — Supporting Information 

# Mechanical transduction of cytoplasmic-to-transmembrane-domain movements in a hyperpolarization-activated cyclic nucleotide–gated cation channel

## Supporting Information

- Supporting information Figure 2 - Displacements of S1 to S6 helices of the TMPC (a-f) in top and bottom view after perturbing the &#x201C;elbow&#x201D; of the C-Linker from the most realistic perturbation direction. The corresponding helix is highlighted and labeled in lime green and the displacement is visualized as yellow arrows. For clarity only one subunit (residue 94-402) is shown.
- Supporting information 4 - K-means clustering of the LRT null model with maximal within-cluster sum of squares (withinss) as a function of number of clusters. The most drastic drop is from three to four clusters.
- Supporting information Figure 1: - Perturbation of the ANM from HCN1 in the cAMP-bound form. The force is applied at the tip of the elbow from the red cluster. This perturbation intends to represent displacements of the cAMP-bound structure in response to cAMP release from the binding site. Note that the red cluster points in the opposite direction of the perturbation from the yellow cluster, which simulates cAMP binding. For clarity, the displacements are only shown for one subunit as in Fig. 2. Color coding of the &#x201C;elbow&#x201D; and the &#x201C;shoulder&#x201D; are the same as in Fig. 1.
- Supplementary Figure 3 - Elastic network model of curated cAMP-free HCN1 structure with a distance cutoff of 13 &#x00C5; for connected residues. The C&#x03B1; atoms of each residue are reduced to spheres (coloring of the subunits as in Fig. 1b) and the connections between atoms are shown as red lines. The elbow domain of the gray subunit is highlighted in light blue and the corresponding shoulder domain in orange as in Fig. 1a.
