# Supplementary material for: Mechanical transduction of cytoplasmic-to-transmembrane-domain movements in a hyperpolarization-activated cyclic nucleotide–gated cation channel
Source: J Biol Chem. 2018 Jun 23;293(33):12908–18. doi: 10.1074/jbc.RA118.002139 (PMC6102142; doi:10.1074/jbc.RA118.002139)
Supplement: Supporting Information [file supp_RA118.002139_135610_2_supp_149079_p9g6mc.docx]

**Supporting information Figure 2**

**
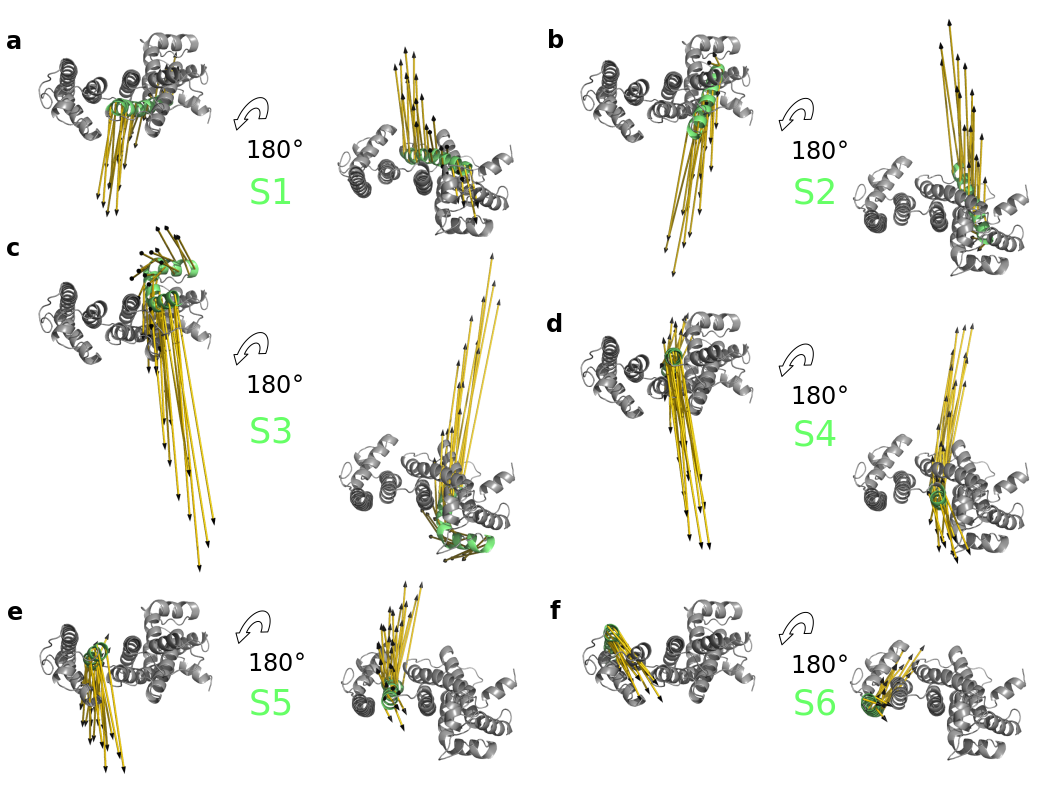
Supporting information Figure 2:** Displacements of S1 to S6 helices of the TMPC (**a-f**) in top and bottom view after perturbing the “elbow” of the C-Linker from the most realistic perturbation direction. The corresponding helix is highlighted and labeled in lime green and the displacement is visualized as yellow arrows. For clarity only one subunit (residue 94-402) is shown.
