# Supplementary material for: Mechanical transduction of cytoplasmic-to-transmembrane-domain movements in a hyperpolarization-activated cyclic nucleotide–gated cation channel
Source: J Biol Chem. 2018 Jun 23;293(33):12908–18. doi: 10.1074/jbc.RA118.002139 (PMC6102142; doi:10.1074/jbc.RA118.002139)
Supplement: Supporting Information [file supp_RA118.002139_135610_2_supp_149077_p956mc.docx]

**Supporting information Figure 3**


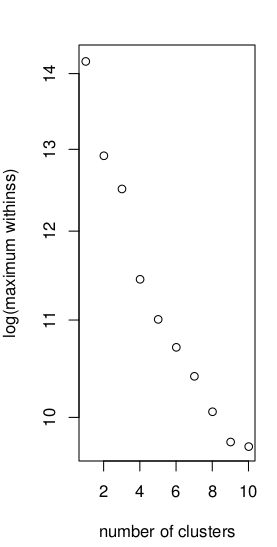


**Supporting information Figure 3:** *K*-means clustering of the LRT null model with maximal within-cluster sum of squares (withinss) as a function of number of clusters. The most drastic drop is from three to four clusters.
