# Supplementary material for: Mechanical transduction of cytoplasmic-to-transmembrane-domain movements in a hyperpolarization-activated cyclic nucleotide–gated cation channel
Source: J Biol Chem. 2018 Jun 23;293(33):12908–18. doi: 10.1074/jbc.RA118.002139 (PMC6102142; doi:10.1074/jbc.RA118.002139)
Supplement: Supporting Information [file supp_RA118.002139_135610_2_supp_149092_p9979t.docx]

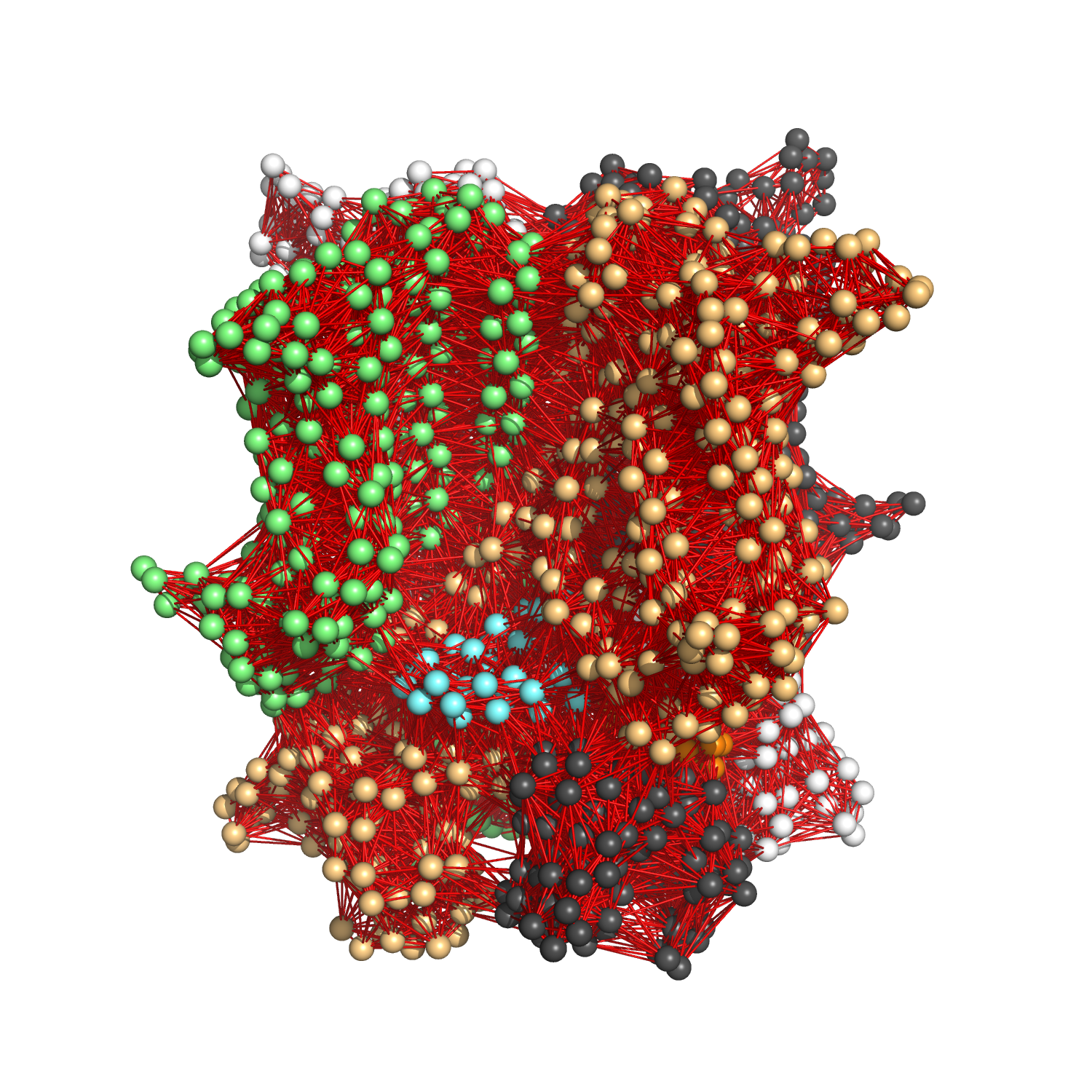


**Supplementary Figure 3:** Elastic network model of curated cAMP-free HCN1 structure with a distance cutoff of 13 Å for connected residues. The Cα atoms of each residue are reduced to spheres (coloring of the subunits as in Fig. 1b) and the connections between atoms are shown as red lines. The elbow domain of the gray subunit is highlighted in light blue and the corresponding shoulder domain in orange as in Fig. 1a.
